# Assessment of Clinical Outcomes and Quality of Life Following Laminectomy and Lateral Mass Screw Fixation in Patients With Cervical Myelopathy

**DOI:** 10.7759/cureus.88311

**Published:** 2025-07-19

**Authors:** Jagdeep Singh, Ajaybir Singh, Rajdeep Sandhu, Albert Lalhmunsanga, Raj Bahadur

**Affiliations:** 1 Department of Orthopaedics, Guru Gobind Singh Medical College and Hospital, Faridkot, IND; 2 Department of Orthopaedics, Government Medical College, Amritsar, Amritsar, IND; 3 Department of Orthopaedics, Regional Spinal Injury Centre, Mohali, IND

**Keywords:** degenerative cervical myelopathy, lateral mass screw, multilevel cervical spondylosis, posterior spinal fixation and fusion, whiplash injury

## Abstract

Introduction: Degenerative cervical myelopathy is a progressive neurological disorder resulting from extradural compression of the spinal cord at the cervical level. Clinically, it manifests with motor dysfunction, including hand clumsiness, gait instability, and signs of upper motor neuron involvement. In patients with multilevel cervical involvement, posterior decompressive laminectomy combined with lateral mass screw fixation is aimed at achieving effective spinal cord decompression while maintaining postoperative spinal stability.

Objective: This prospective case series aims to evaluate the clinical outcomes and quality of life following posterior decompression and lateral mass fusion in patients with multilevel compressive cervical myelopathy.

Materials and methods: A total of 30 patients with compressive cervical myelopathy involving two or more levels were included in the study. Radiological assessments, including X-rays, MRI, and CT scans, were performed for evaluation. All patients underwent posterior cervical decompressive laminectomy combined with lateral mass fusion. Functional outcomes and quality of life were assessed preoperatively and at the one-year postoperative mark using the Japanese Orthopaedic Association (JOA) score and the Denis Pain and Work Scale as primary outcomes. Secondary outcomes assessed were perioperative complications, such as blood loss, neurological deterioration, infections, and implant failure.

Results: The mean age of the patients was 49.5 years. Surgical intervention involved two-level decompression in nine patients (30%), three-level decompression in 14 patients (46%), and four-level decompression in seven patients (24%). The average blood loss was 202.6 mL, and the mean operative time was 135.3 minutes. At the one-year follow-up, the modified Japanese Orthopaedic Association (mJOA) score improved from a preoperative mean of 7.3 to 12.4. The Denis Pain Scale showed an improvement from 3.9 to 1.8, while the Denis Work Scale improved from 4.8 to 3.2. Postoperative complications included wound infections in two patients and transient neurological worsening in four patients, all of which resolved over time. There were no instances of implant failure.

Conclusion: Multilevel cervical laminectomy combined with lateral mass screw fixation is a reliable and effective surgical approach for the management of multilevel cervical myelopathy. This technique facilitates adequate spinal cord decompression, minimizes the risk of postoperative spinal deformities, and preserves the structural integrity of the posterior tension band.

## Introduction

Degenerative cervical myelopathy is characterized by extradural compression of the spinal cord at the cervical level, leading to a constellation of neurological deficits, including spasticity, muscle weakness, hyperreflexia, hand clumsiness, and gait disturbances. The condition typically presents with insidious onset and progresses in a stepwise fashion, ultimately resulting in functional deterioration [1].

The natural history of cervical myelopathy has been described in early observational studies, notably by Nurick. He found that individuals with initially mild symptoms often experienced minimal or no significant clinical deterioration over time. Conversely, those who presented at an older age or with more advanced neurological deficits demonstrated greater functional decline. The poorest outcomes were observed in elderly patients with severe disability at presentation, highlighting the prognostic value of both symptom severity and patient age [2].

The primary objectives of surgical intervention are to decompress the spinal cord, stabilize the spinal column, and restore physiological sagittal alignment. Surgery is often necessary to prevent further neurological deterioration, with anterior or posterior decompression, frequently combined with spinal fusion, constituting the mainstay of treatment.

In cases where the compressive pathology originates from anterior structures, such as disc-osteophyte complexes or ossified posterior longitudinal ligament (OPLL), posterior techniques enable indirect decompression. This is achieved by expanding the spinal canal, allowing the spinal cord to migrate posteriorly away from ventral compressive elements. Effective posterior translation of the cord depends on maintaining a neutral or lordotic cervical sagittal alignment, which facilitates this decompressive shift [3].

Decompressive laminectomy is performed at the junction between the laminae and the lateral masses to alleviate spinal cord compression. In patients presenting with concurrent radicular symptoms, a foraminotomy may be performed simultaneously to address associated foraminal stenosis. The incorporation of lateral mass screw fixation enhances biomechanical stability, resulting in a rigid construct that facilitates spinal alignment and has been shown to improve fusion rates [4].

Wada et al. emphasized the importance of integrating clinical presentation, functional assessment, and imaging findings when determining the indication for surgical intervention in cervical myelopathy. They advocated for operative management in patients exhibiting symptoms such as impaired gait and reduced fine motor coordination, particularly when accompanied by a Japanese Orthopaedic Association (JOA) score below 13 and radiological evidence of spinal cord compression [5].

Areas of maximal spinal cord compression often appear as regions of increased signal intensity (ISI) on T2-weighted magnetic resonance imaging (MRI), commonly indicative of myelomalacia. These changes are believed to reflect underlying pathological processes such as spinal cord edema or irreversible neuronal injury. The presence of such signal alterations has been correlated with more severe neurological impairment and is generally considered a negative prognostic factor for postoperative functional recovery [6].

## Materials and methods

This study included 30 patients diagnosed with compressive cervical spondylotic myelopathy (CSM) who underwent cervical laminectomy and lateral mass fusion involving two or more levels. Preoperative radiological evaluations of the cervical spine were performed using X-rays, computed tomography (CT) scans, and MRI. Neurological status was assessed preoperatively utilizing the JOA score, as well as the Denis Work and Pain Scales.

Postoperative clinical evaluations were conducted immediately following surgery, while radiological assessments were performed prior to discharge and subsequently at two weeks, three months, six months, and 12 months post-surgery. All patients completed a final follow-up at one year. The primary outcomes of clinical and neurological improvement were measured using the JOA score and the Denis Work and Pain Scales. Complications, including neurological deterioration, neck pain, and brachialgia, as secondary outcomes, were systematically recorded and analyzed.

Ethical approval for the study was obtained from the Institutional Ethics Committee, and written informed consent was secured from all participants.

Inclusion criteria

The study included patients diagnosed with CSM and spinal cord compression involving at least two levels. The underlying etiologies comprised degenerative cervical spondylosis, OPLL, ossification of the ligamentum flavum (OLF), and traumatic whiplash injury superimposed on a pre-existing spondylotic cervical spine.

Exclusion criteria

Patients with cervical spine infections, tumors, pathological conditions (e.g., tuberculosis, rheumatoid arthritis), traumatic cervical fractures or dislocations, gross cervical instability (congenital instability or significant movement on dynamic X-rays), and non-compressive myelopathy were excluded from the study.

Surgical procedure

The posterior cervical laminectomy and lateral mass screw fixation were performed with the patient in the prone position under general anesthesia. A posterior midline incision was made along the cervical spine, followed by meticulous dissection of the fascia and paravertebral musculature bilaterally from the spinous processes to fully expose the lateral mass surfaces. Lateral mass screws were inserted on either side of the planned levels, and the screw position was confirmed under fluoroscopy.

A 3-5 mm wide slot was created at the lamina-facet junction using a high-speed burr, extending to the inner cortex. This was followed by meticulous removal of the inner cortex with a 1-2 mm lamina rongeur to complete the slot. The procedure was then repeated contralaterally using the same technique. Magnification was employed as necessary to enhance visualization. The spinous process was gently pulled to elevate the bone en bloc from the spinal canal using Kocher forceps. Complete detachment of the ligamentum flavum from the underlying dura was achieved using rongeurs and a Penfield dissector.

In cases where patients presented with nerve root symptoms, foraminal decompression was performed to relieve neural impingement. A connecting rod and horizontal connectors were then installed, and the optimal placement of the internal fixation was confirmed using C-arm fluoroscopy. Finally, bone grafts were applied bilaterally to facilitate bone fusion.

## Results

Age and gender distribution

A total of 30 patients were included in the study, with a mean (SD) age of 49.5 (3.85) years. The highest proportion of patients, 10 (33.3%), were in the 41-50 year age group, followed by six (20%) in the 51-60 year group. Other age distributions included five patients (16.7%) aged 71-80 years, four (13.3%) aged 61-70 years, three (10%) aged 31-40 years, and two (6.7%) aged 18-30 years (Table 1).

**Table 1:** Age distribution of patients SD: standard deviation

Age (Years)	Number	Percentage (%)
18 to 30	2	6.67
31 to 40	3	10
41 to 50	10	33.33
51 to 60	6	20
61 to 70	4	13.33
71 to 80	5	16.67
Total	30	100
Mean	49.5	-
SD	3.85	-

Most of the cohort was male, comprising 23 patients (76.67%), while seven patients (23.33%) were female.

Etiology and levels involved

Regarding the etiology of compressive cervical myelopathy, OPLL was observed in nine cases (30%), non-traumatic multilevel degenerative CSM in eight cases (26.67%), and degenerative CSM associated with whiplash injury in 13 cases (43.3%) (Table 2).

**Table 2:** Distribution of cases according to etiology OPLL: ossified posterior longitudinal ligament; CSM: cervical spondylotic myelopathy

Etiologic Profile	Number	Percentage (%)
OPLL	9	30
Multilevel degenerative CSM	8	26.67
Whiplash injury in degenerative CSM	13	43.33
Total	30	100

Surgical intervention involved two-level decompression in 30% of cases, three-level in 46%, and four-level in 23%, with an average blood loss of 202.6 mL and a mean operative time of 135.3 minutes.

Clinical outcome and complications

Functional recovery was noted, as evidenced by improvements in the JOA score, which improved from a preoperative mean of 7.3 to 12.4 at one year. Incremental improvements were observed at interim assessments, 7.9 at two weeks, 9.1 at three months, 10.2 at six months, and 10.9 at nine months (Table 3).

**Table 3:** Longitudinal assessment of neurological recovery using the JOA score * p-value < 0.05 was considered statistically significant. Statistical analysis was performed using repeated measures ANOVA. ANOVA: analysis of variance; JOA: Japanese Orthopaedic Association

JOA Score	Preoperative	2 Weeks	3 Months	6 Months	9 Months	12 Months	P-value (Repeated Measures ANOVA)
Mean	7.3	7.9	9.1	10.2	10.9	12.4	0.003*
Standard deviation (SD)	1.2	2.6	3.1	3.2	2.6	2.9	-

Pain outcomes, assessed using the Denis Pain Scale, also demonstrated marked improvement, with scores decreasing from a preoperative mean of 3.9 to 1.8 at the one-year follow-up (Table 4).

**Table 4:** Longitudinal assessment of pain using the Denis Pain Scale * p-value < 0.05 was considered statistically significant. Statistical analysis was performed using repeated measures ANOVA. ANOVA: analysis of variance

Time Point	Mean Score	Standard Deviation (SD)	P-value (Repeated Measures ANOVA)
Preoperative	3.9	1.3	0.003*
2 Weeks	3.1	1.1	-
4 Weeks	2.6	0.9	-
6 Months	2.4	0.9	-
9 Months	2.2	0.8	-
12 Months	1.8	0.3	-

Similarly, work capacity, evaluated through the Denis Work Scale, improved from a baseline score of 4.8 to 3.2 at one year, reflecting enhanced functional independence (Table 5).

**Table 5:** Longitudinal assessment of work capacity using the Denis Work Scale * p-value < 0.05 was considered statistically significant. Statistical analysis was performed using repeated measures ANOVA. ANOVA: analysis of variance

Time Point	Mean Score	Standard Deviation (SD)	P-value (Repeated Measures ANOVA)
Preoperative	4.8	1.2	0.004*
2 Weeks	4.5	1.3	-
4 Weeks	4.1	0.8	-
6 Months	3.9	0.9	-
9 Months	3.6	0.7	-
12 Months	3.2	0.3	-

Muscle strength improvements in both upper and lower limbs were statistically significant over the one-year follow-up (Tables 6, 7), underscoring the efficacy of the surgical intervention (Tables 6, 7).

**Table 6:** Comparison of preoperative and one-year postoperative upper limb function * p-values < 0.05 were considered statistically significant. Palmar grip was assessed on an ordinal scale and analyzed using the Wilcoxon signed-rank test. All other variables were analyzed using the paired t-test for continuous data.

Upper Limb Joint	Side	Preoperative (Mean ± SD)	1-Year (Mean ± SD)	P-value (Statistical Test)
Shoulder	Left	2.30 ± 0.80	3.46 ± 1.10	0.01* (Paired t-test)
	Right	2.37 ± 0.70	3.83 ± 1.00	0.03* (Paired t-test)
Elbow	Left	2.87 ± 0.90	3.71 ± 1.10	0.04* (Paired t-test)
	Right	2.25 ± 0.80	3.46 ± 1.20	0.00* (Paired t-test)
Wrist	Left	2.87 ± 0.80	3.48 ± 1.20	0.00* (Paired t-test)
	Right	2.61 ± 0.80	3.37 ± 1.20	0.00* (Paired t-test)
Palmar Grip	Left	Poor (ordinal scale)	Moderate (ordinal scale)	0.008* (Wilcoxon signed-rank test)
	Right	Poor (ordinal scale)	Moderate (ordinal scale)	0.006* (Wilcoxon signed-rank test)

**Table 7:** Comparison of preoperative and one-year postoperative lower limb function * p-values < 0.05 were considered statistically significant. All comparisons were conducted using the paired t-test for continuous data.

Lower Limb Joint	Side	Preoperative (Mean ± SD)	1-Year (Mean ± SD)	P-value (Paired t-test)
Hip	Left	1.75 ± 0.80	3.96 ± 1.20	0.02*
	Right	1.37 ± 0.70	3.71 ± 1.20	0.00*
Knee	Left	2.37 ± 0.90	3.89 ± 1.10	0.02*
	Right	2.56 ± 0.80	3.97 ± 1.30	0.04*
Ankle	Left	2.37 ± 0.80	3.65 ± 1.10	0.00*
	Right	2.75 ± 0.90	3.90 ± 1.20	0.03*

The overall complication rate was low. Transient dysphagia occurred in four patients (13.33%), all of whom recovered within two to four weeks with conservative management. Superficial wound infections were noted in two patients (6.67%); one case required surgical debridement, while the other was managed successfully with antibiotics. Postoperative neurological deterioration occurred in three patients (10%), all of whom achieved full recovery within three to six months. Notably, no cases of implant failure were reported during the follow-up period (Table 8).

**Table 8:** Complications

Complications	Number	Percentage (%)
Transient dysphagia	4	13.33
Post-operative neurological symptom deterioration	3	10
Screw pullout	0	0
Wound infection	2	6.67

## Discussion

Degenerative changes are manifested in the cervical spine as disc height loss, facet and uncovertebral joint osteophytes, OPLL, and hypertrophic ligamentum flavum. Neural compression from these structures may lead to the development of clinical symptoms of myelopathy [1].

In these patients, a common subjective complaint is the perception of a “dysfunctional hand,” often accompanied by a noticeable decline in fine motor skills, handwriting, and manual dexterity. Clinical examination frequently reveals atrophy of the thenar and hypothenar eminences, weakness in wrist extensor muscles, and impaired thumb opposition. Gait and balance disturbances typically occur due to proximal lower extremity weakness. Additional clinical signs of myelopathy include hyperreflexia, clonus, pathological reflexes such as bilateral Babinski signs, and sensory deficits including loss of proprioception, stereognosis, and diminished pinprick and vibratory sensation [1]. In severe cases, or those presenting with a whiplash injury in pre-existing cervical spondylosis with narrowing of the spinal canal, may present with overt paralysis, potentially involving bowel and bladder dysfunction [1].

While anterior cervical discectomy and fusion are considered the preferred treatment for single-level cervical myelopathy, the optimal approach for patients with multilevel involvement is debatable. Fang et al. retrospectively compared enlarged laminectomy with lateral mass screw fixation (EL-LMSF) and anterior cervical decompression and fusion (ACDF) in patients with multilevel cervical myelopathy and radiculopathy (CMR) with kyphosis. EL-LMSF resulted in superior pain relief (lower Visual Analogue Scale (VAS) scores) and greater cervical alignment correction (C2-C7 angle: 10.7° vs. 8.5°). ACDF had a shorter operative time (103 vs. 125 minutes) and less blood loss (78 mL vs. 226 mL). Neurological improvement (JOA scores) was comparable between groups. Notably, EL-LMSF had a lower complication rate, supporting its safety and efficacy as a posterior approach [7].

A retrospective cohort study by Levy et al. demonstrated that prolonged preoperative symptom duration, particularly exceeding two years, was significantly associated with reduced postoperative improvement in both functional outcomes and symptom-specific measures following posterior cervical decompression and fusion (PCDF). In contrast, patients who underwent surgery within six months of symptom onset experienced the greatest gains in neurological function and disability reduction. Notably, improvement in pain scores was observed across all symptom duration groups, indicating that posterior decompression remains effective for alleviating nociceptive and radicular pain, even in the setting of chronic symptoms [8].

The presence of T2-weighted ISI with multilevel degenerative cervical myelopathy (MDCM) on MRI has long been associated with more severe spinal cord damage and worse postoperative neurological recovery, making the surgical strategy critical.

Qu et al. conducted a retrospective, propensity-matched study comparing posterior-only and one-stage combined posteroanterior decompression in multilevel cervical myelopathy with T2-weighted ISI over an eight-year follow-up. While the combined approach offered better short-term neurological recovery, long-term outcomes were similar between groups in terms of JOA and Neck Disability Index (NDI) scores. However, it was associated with significantly greater operative time, blood loss, and procedure-specific complications like CSF leakage and dysphagia. These findings highlight the need to individualize surgical strategies based on disease severity, patient comorbidities, and long-term functional goals [9].

Nguyen et al. conducted a STROCSS-compliant retrospective cohort study comparing laminoplasty (LP) and laminectomy with posterior spinal fusion (LPSF) in 65 patients with multilevel CSM (≥12-month follow-up; LP = 31, LPSF = 34). Both groups showed similar neurological and pain outcomes based on JOA and VAS scores. However, LPSF was more appropriate for patients with cervical kyphosis, instability, or severe mechanical pain, offering greater structural correction (mean Cobb angle: 14.15° vs. 9.35°). This came with longer operative time and higher complication rates (8.6% vs. 3.2%). Surgical approach should be guided by preoperative alignment, spinal stability, and axial pain, with LP preferred for motion preservation and LPSF for structural correction [10].

In a single-center retrospective study, Chen et al. reported a 25% rate of instrumentation failure following ≥ four-level laminectomy and posterior cervical fusion (PCF). Identified risk factors included osteoporosis (57.1% in patients with osteoporosis vs. 14.3% in those without; p = 0.001), fixation terminating at the C7 vertebra (36.3% vs. 9% when ending at T1; p = 0.019), and low regional bone quality on CT scans indicated by decreased Hounsfield unit (HU) values along screw trajectories. Importantly, all cases of instrumentation failure in this study were asymptomatic, with no associated neurologic deficits or need for revision surgery [11].

In a multicenter prospective study, Nakashima et al. conducted a propensity score-matched analysis to compare the clinical outcomes of LP and posterior fusion (PF) in the surgical management of cervical OPLL. At two-year follow-up, both techniques yielded comparable results in neurological recovery and health-related quality of life. However, PF was more effective in limiting the radiographic progression of OPLL, indicating its potential advantage in patients with extensive ossification or adverse anatomical characteristics [12]. These findings emphasize the need for a patient-specific approach to surgical planning, guided by clinical presentation and imaging features, to optimize outcomes in cervical OPLL.

The study by Singrakhia et al. supported multilevel cervical laminectomy with selective lateral mass screw fixation as a safe and effective approach for managing multilevel CSM in patients with neutral or lordotic alignment. This approach resulted in significant neurological recovery, maintained sagittal alignment, and avoided common complications associated with both anterior and non-instrumented posterior surgeries. Selective instrumentation, rather than routine full-segment fixation, appeared sufficient in appropriately selected patients, offering a cost-effective and biomechanically sound alternative. Preservation of sagittal balance and the absence of postoperative kyphosis further reinforced the value of instrumented fusion [13].

In patients undergoing posterior cervical laminectomy and fixation for multilevel cervical degenerative myelopathy (CDM), the addition of C7-T1 transfacet fixation has been shown to significantly reduce the T1 slope, thereby enhancing sagittal stability. This restoration of sagittal balance correlates with improved neurological outcomes in both short- and long-term follow-up periods [14].

In a retrospective clinical and radiographic study, Du et al. evaluated the outcomes of a posterior surgical approach in patients with multilevel CDM associated with kyphotic deformity. The surgical technique involved an enlarged laminectomy, entailing removal of the inner edges of the facet joints and decompression of the neural foramina, combined with lateral mass screw fixation using the Magerl technique and autologous bone grafting. The authors reported this approach to be both effective and safe, demonstrating substantial neurological improvement, significant restoration of cervical lordosis, and a notably low complication rate. Importantly, no cases of C5 nerve root palsy were observed in the cohort, underscoring the potential of this technique to minimize common postoperative complications [15]. Similarly, in our study, none of the cases suffered a C5 palsy.

In a prospective, randomized controlled trial conducted at a U.S. academic institution, Manzano et al. compared clinical, radiographic, and patient-reported outcomes between expansile cervical laminoplasty (ECL) and cervical laminectomy with fusion (CLF) in the management of multilevel CSM. Despite the limited sample size of 16 adult patients, the study demonstrated that ECL was associated with favorable outcomes, particularly in preserving cervical range of motion and improving functional status. These findings suggest that ECL may serve as a viable alternative to CLF in appropriately selected patients, especially in cases where the preservation of cervical mobility is a key surgical objective [16].

In a comprehensive systematic review, Yang et al. evaluated 75 studies involving over 19,500 patients to assess posterior surgical strategies for MDCM. The analysis revealed considerable heterogeneity in study design, patient selection, and outcome reporting, with most studies being retrospective and single-center. Fusion techniques appeared more effective in limiting OPLL progression but were associated with higher morbidity. LP demonstrated comparable clinical outcomes to laminectomy alone, with potential benefits in preserving cervical alignment [17]. However, the absence of high-quality evidence and consistent reporting precludes definitive conclusions on the optimal surgical approach.

Limitations and future directions

A key strength of this study lies in its prospective design. However, notable limitations include the absence of a control group and the lack of randomization, which may introduce bias and restrict the generalizability of the findings. Future studies employing randomized controlled methodologies with longer follow-up periods are warranted to confirm and build upon these results.

## Conclusions

The primary surgical goal in managing multilevel cervical myelopathy is to achieve adequate decompression while preserving cervical spine stability. Results of our study and recent literature suggest that multilevel cervical laminectomy combined with lateral mass screw fixation is a safe and effective surgical approach for addressing multilevel compressive cervical myelopathy. This technique effectively decompresses the spinal cord, reduces the risk of spinal deformities, and preserves the integrity of the posterior tension band, thereby improving cervical alignment and functional outcomes. In contrast, procedures such as LP or non-instrumented laminectomy may compromise these benefits. Even the anterior procedures in multilevel cases are associated with increased risk of dysphagia, pseudarthrosis, and implant failures.
